# Train Strategies for Haptic and 3D Simulators to Improve the Learning Process in Dentistry Students

**DOI:** 10.3390/ijerph19074081

**Published:** 2022-03-30

**Authors:** Gleyvis Coro-Montanet, María Jesús Pardo Monedero, Julia Sánchez Ituarte, Ana de la Hoz Calvo

**Affiliations:** Preclinical Dentistry Department, School of Biomedical and Health Sciences, Villaviciosa de Odoón Campus, Universidad Europea de Madrid, 28670 Madrid, Spain; gleyvis.coro@universidadeuropea.es (G.C.-M.); mariajesus.pardo@universidadeuropea.es (M.J.P.M.); julia.sanchez@universidadeuropea.es (J.S.I.)

**Keywords:** Simodont^®^, procedural training, simulator briefing

## Abstract

Dental training faces the growing shortage of extracted teeth and the ethical precepts of Bionot learning on patients and reducing the environmental damage that preclinical trainings generate. Haptic and 3D simulators reproduce pathologies and provide a greater magnification of the processes, reducing water expenditure and pollution, but their curricular integration is complex. Two resources of complementary use (informative written manual and video tutorial) were designed to facilitate the theoretical and technical domain (know how the simulator works and make it work), as well as the advanced management of the simulator (operate the simulator autonomously, without setbacks). After 5 years of using these resources, an evaluative study was conducted with 175 students and 32 teachers. The aim was to assess the student’s perception of knowledge/learning, its statistical relationship with the didactic resources used and compare these results with the teachers’ perception of their students’ knowledge/learning. Spearman’s Rho coefficient and Kolmogorov-Smirnov test were performed. Both students and teachers considered that the technical domain (make the simulator work) was the domain that prevailed the most. There was a tendency for students not to value much the necessity of a specific preparation prior to using the simulator. This tendency resulted in a low level of study of both the written manual and the video tutorial. In conclusion, both students and teachers considered that the best strategy of knowledge/learning was the direct exchange with the simulator.

## 1. Introduction

Historically, traditional procedural simulators linked to rotating instruments have been used in undergraduate dentistry training. These allow students to train gross and fine motor skills [1,2,3]. The phantom head has been used in the dental academy since Oswald Fergus introduced it in 1894 [4], being the most used and widespread method so far. However, all this is not without causing environmental damage, waste of water and pollution during the drilling of synthetic structures or extracted natural teeth.

The technological development of recent times has demonstrated the need for the learning of the basic skills of dentistry to be supported by more modern resources and simulators [5,6].

Medicine was the first clinical specialty to be favored, since the year 2000, with the introduction of haptic technology using tactile devices. These allowed to reproduce with realism the operative sensitivity, with greater safety for the patient and a reduction of the environmental damage [7,8,9]. Almost at the same time, dentistry also introduced devices that allowed to control the amount of force that the operator had to apply during the treatment of the patient and combine it with mixed realities [10,11,12,13].

Haptic technology allows the operator to feel and manipulate tools and organs in a low-risk virtual environment, as well as perform tasks such as cutting soft and hard tissues with high tactile realism [14].

PerioSim^©^ is described as the first dental simulator of virtual reality and haptic technology that, since 2006, allows the student to train in the diagnosis and treatment of periodontal diseases [15].

Later, in 2010, Moog Industrial Group, of Amsterdam, built the dental homework trainer named Simodont^®^. At the same time, the Academic Centre for Dentistry in Amsterdam (ACTA) developed its courseware [16].

In recent years, dental trainer Simodont^®^ has been acquired by Nissin Dental Product Inc, the Japanese manufacturer of dental training and simulation systems. Its manufacture has been extended to 571 units, distributed in 117 dental schools in Asia, Europe, America and Oceania. However, despite the number of units distributed, the introduction into the institutions’ programs as main modules has not been easy [17].

In the evidence found, the use of this type of tools with digital benefits focuses on its use as an unregulated alternative for the students—by voluntary reservation of the student, in tests of admission to the degree, punctual exercises of recovery or reaccreditation of professionals [17,18,19]. However, it is important to publicize and refine the strategies that have consolidated their use in undergraduate studies. Above all, even though it exists a resistance to change, there is a need to adapt to the digital requirements of the new times. The new simulators are a necessity of teaching and are here to stay. They must be improved in their design and the use it is given to them. This is because, as de Boer et al. pointed out [20,21], current dental training faces a major problem in relation to the growing shortage of extracted teeth and the ethical conflict of learning to perform dangerous operations directly on patients.

**Figure 1:**
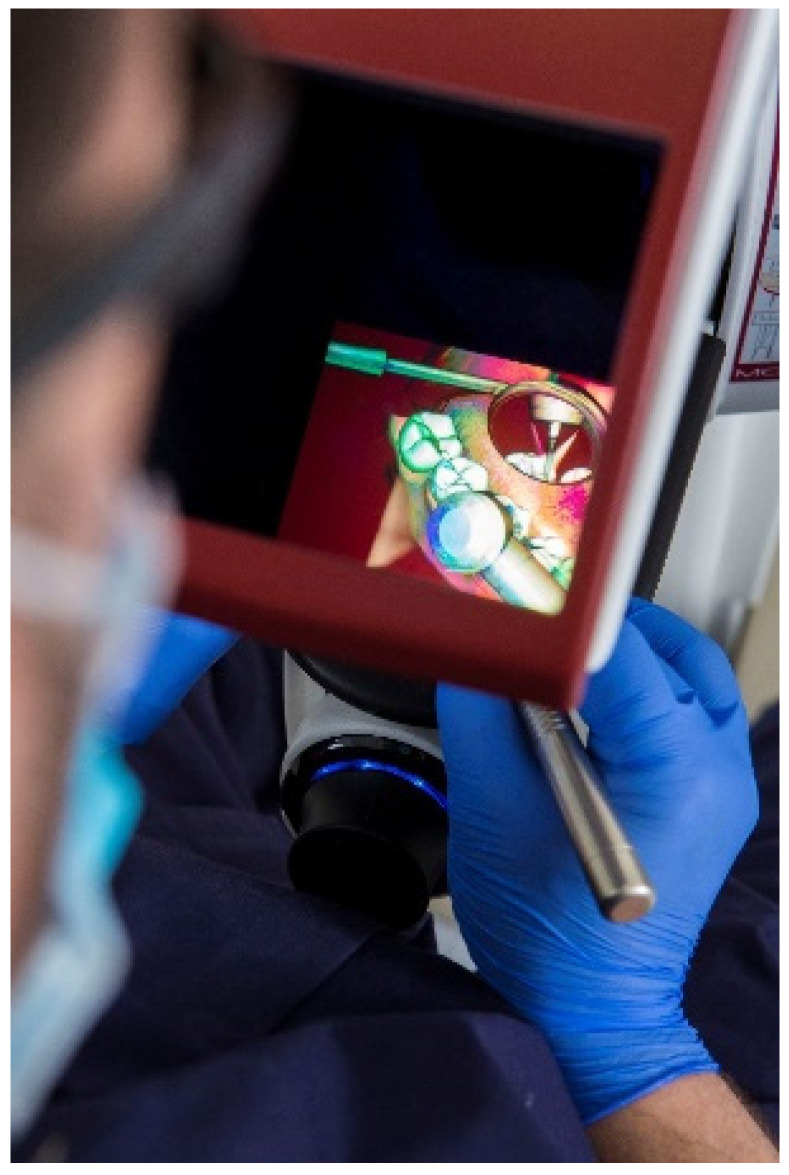
Simodont^®^ dental trainer. Source: Courtesy of the authors. Written permission of NISSIN Dental.

Simodont^®^ (Figure 1) is a recent device, different from the usual task simulators, which has interesting benefits for teaching dentistry. It contemplates the ability to reproduce pathologies and the contribution of a greater magnification [16]. This supposes a learning and integration task that teachers and students find difficult to assume.

At the Universidad Europea de Madrid (UEM), a specific methodology was developed to integrate these simulators into the curriculum [22]. For its creation, the taxonomies of active learning, Harris’ Technological Pedagogical Content Knowledge (TPACK) integration model [23] and the best scientific evidence of simulation-based learning applied to the development of competences through task simulators were considered [24].

A team of experts in active learning from the UEM created two didactic resources designed to facilitate students and teachers the proper use and control of the Simodont^®^.

**Figure 2:**
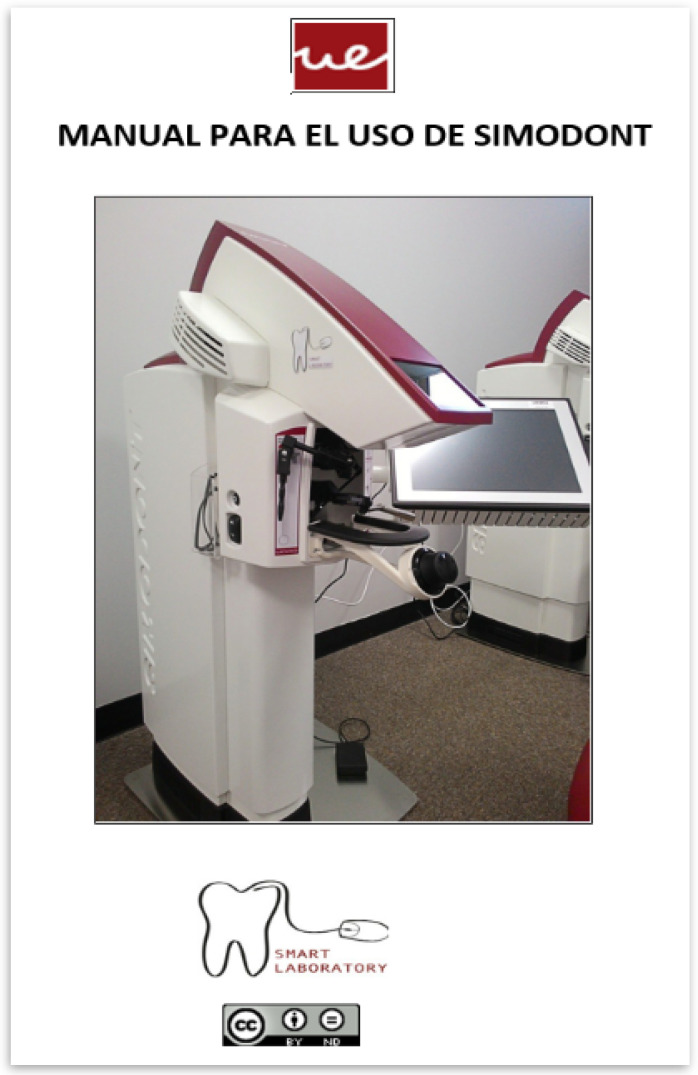
Simodont^®^ infographic manual. Source: Courtesy of the author. Written permission of NISSIN Dental.

**Figure 3:**
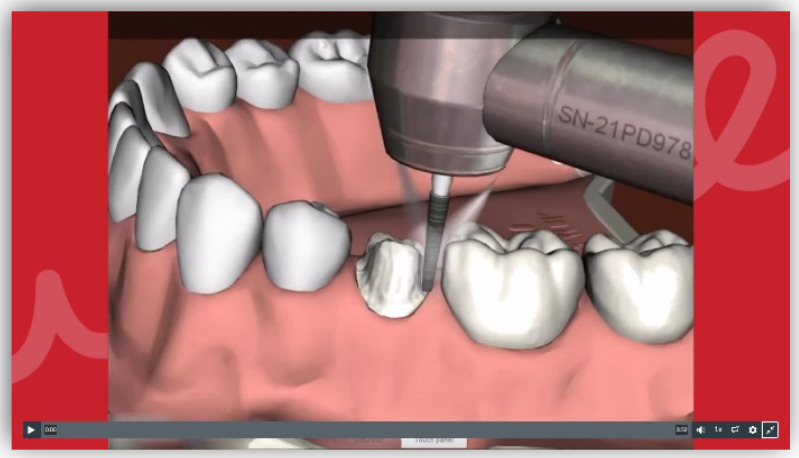
Video tutorial frame. Source: Courtesy of the authors. Written permission of NISSIN Dental.

These resources were:

1. Simodont^®^ informative written manuals (Figure 2);
2. The video tutorials of Simodont^®^ software management (Figure 3).

The informative written manuals included a detailed explanation of the simulator hardware and software. These explained the ergonomics and how to use the interfaces and elements of the equipment through diagrams, photos and short texts. Its full reading would consume about 30 min.

The video tutorials focused on the description of the management of the software and courseware in less than 10 min.

These resources were designed based on the idea of improving learning efficiency, quickly transmitting key notions, based on the concept that focused and concise material helps the student to better integrate the content [25,26].

Both were elaborated with a screen capture software and in accordance with the didactic precepts that describe the literature for the construction of these tools [27].

Specific skills workshops were created from the digital library of the simulators using this methodology (use of both the written manual and video tutorials) as a support. These skills workshops constituted a compulsory module in six different subjects (Introduction to the clinic, Biomaterials, Paediatric Dentistry I and II, Restorative III and IV and Prosthodontics III and IV) for a period of five years (from 2015 to 2020). In this way, the use of the haptic equipment became permanent in the dental school of the UEM.

## 2. Materials and Methods

The research team’s objectives were to evaluate the students’ perception of their learning in the use of the haptic and 3D simulator at 3 different levels. These levels were: theoretical domain (knowing how it works), technical domain (how to make it work) and advanced knowledge domain (operating the simulator autonomously and smoothly). Once this had been evaluated, their statistical correlation with the didactic resources used was analyzed. The students’ perception of knowledge/learning (as assessed by themselves and by their teachers) was also compared.

For the evaluative study, two samples were selected from the UEM teaching and student populations. Those selected had to have used Simodont^®^ with the didactic resources described above (written information manuals and video tutorials).

Overall, 175 students were randomly selected from among the students of 3rd and 4th year of the Degree in Dentistry at the UEM that had used Simodont during their preclinical practices. It was stablished as an inclusion criterion to have carried out more than 3 skill workshops with the simulators with the didactic resources designed for the integration of these activities. Those who did not fulfill this minimum number and condition were excluded from the sample.

**Figure 4:**
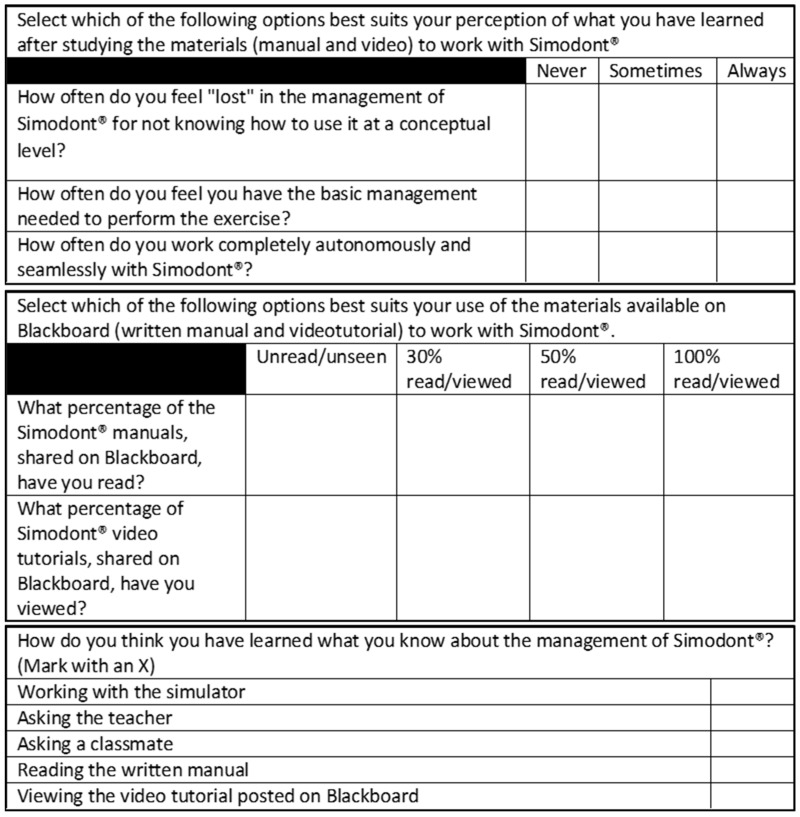
Questionnaire for students.

In the sample of students, the perception of individual knowledge/learning was studied in terms of the theoretical, technical and advanced management domains, and the reading of the informative written manual and the visualization of the video tutorial were measured. This was performed by using the questionnaire shown in Figure 4.

**Figure 5:**
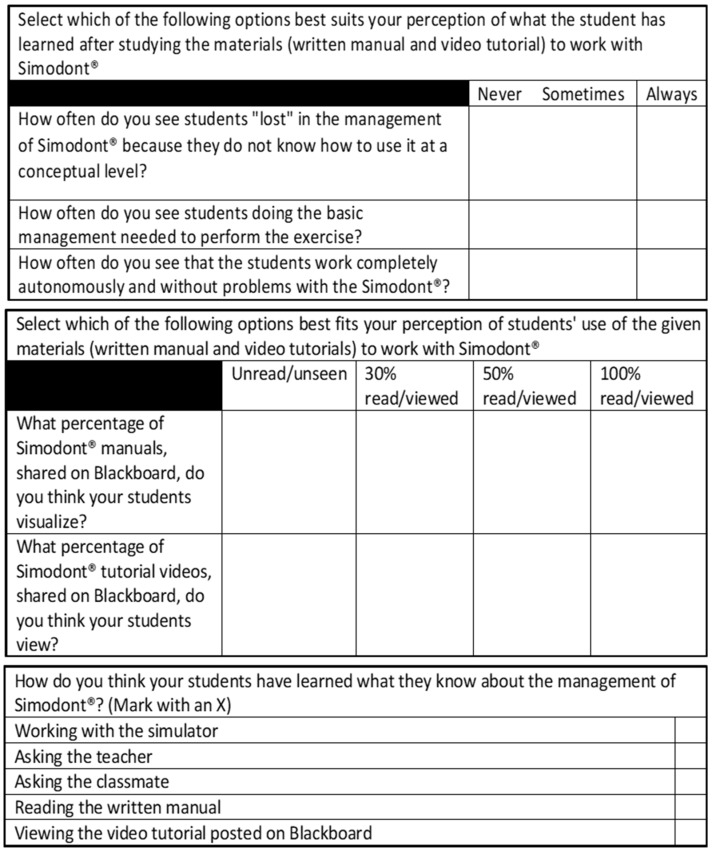
Questionnaire for teachers.

The sample of teachers was composed by all the teachers that had used the Simodont for their preclinical practices during those 5 years. In this sample, the teacher’s perception of global knowledge/learning about the students was studied, objectified and evaluated with the same variables (theoretical, technical and advanced management domains of the simulator). Figure 5 shows the questionnaire used for this purpose.

The data collection tools for both samples were questionnaires with polytomous closed questions on the variables described on Table 1 and Table 2.

At the end of the questionnaire, a closed polytomous question was introduced to collect the perception of the respondents (students and teachers) on the different strategies of knowledge/learning of the complex simulator. In this question they had to select which options did they considered to have been the most effective for their learning. Not only reading the manual or watching the videos were considered as options. Other strategies were also measured, such as direct work with the simulator, questions to the teacher and questions to other classmates (Figure 4 and Figure 5).

For the collection of data, the ethical precepts were always respected. All participants in the research agreed to participate voluntarily, the questionnaires were anonymized and coded for blind use, in accordance with data protection laws.

A briefing was held prior to the survey to reduce biases due to misinterpretation, clarifying that the perception of knowledge/learning was related only to the use of the simulator and not their performance on the skills workshops. The culture of measuring curricular learning (the contents and skills directly linked to the learning objectives) was a limitation of this study, which had the objective of evaluating only the domain that the student had of the simulator. To minimize this bias, only enrolled students who had access to the simulator briefing (information and previous training to achieve the best management of the equipment) were surveyed. This reduced the size of the sample, without affecting the significance of it.

Once the results were obtained, they were treated in the statistical program SPSS v23 and processed using descriptive statistics. For the study of the variables contemplated, the Kolmogorov–Smirnov test was performed, rejecting the null hypothesis test, in all cases, since the asymptotic significance for the studied variables was less than 0.05, with *p* = 0.00. So, they were considered non-normal variables. Analysis based on the filled questionnaires was performed using Spearman’s Rho correlation method. 

## 3. Results

The theoretical, technical and advanced management domains reflected a medium level (level 2: sometimes) of perception for the surveyed sample. The mean of technical domain was higher (operating the simulator at a basic level that allows the exercise to be carried out). The lowest mean was in the advanced management domain (carry out the exercise autonomously and without setbacks).

Regarding the use of the designed didactic resources, the highest means corresponded to the study of the informative written manual and the video tutorials, both around 50%, considered as a medium level-with large standard deviations, which indicates dispersion in the responses. Table 3 shows the means of the student’s learning perception.

Table 4 shows the same analysis for the sample of the teachers. Teachers assessed the theoretical domain of their students more rigorously (the mean was around 1.97, which offers a low perception of the level of knowledge/learning). It was the lowest standard deviation of the analysis, which shows the least dispersion of the answers and gives greater weight to the data.

In the teacher’s perception, the students’ means of technical domain were like those perceived by the students themselves on the same variable. Meanwhile, teachers show a greater perception of advanced management domain in students than the students themselves, who are self-critical and cautious when it comes to defining themselves as advanced managers.

On the other hand, the means of teacher perception describe less study of the manual (around 30% of reading) and a greater visualization of videos (around 50% of visualization).

Table 5 shows that in the responses of the surveyed students there is no significant correlation between the knowledge/learning domains of the simulator (theoretical, technical and advanced management domain) and the study of the manual and the video. The Spearman’s Rho coefficient shows a very weak correlation (<0.29) and a *p* > 0.05 in all cases. On the other hand, there is a positive correlation between the study of the manual and the study of the video. There is also a correlation between the three domains with each other. However, these correlations are not strong (slightly greater than 0.29) in most cases.

Table 6 summarizes the correlation analysis for the sample of teachers and shows more powerful correlation coefficients (0.570), with significance (0.001) between the variable’s theoretical domain and manual study. Being the most powerful the correlation between the technical domain and the study of video (0.901) with *p* = 0.000 and between technical and advanced management domains (correlation coefficient of 0.544 and *p* = 0.001).

**Figure 6:**
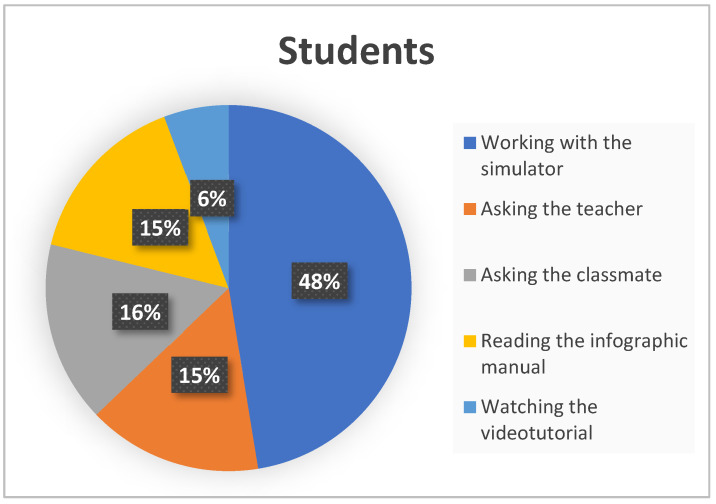
Percentages of the most effective strategies for knowledge/learning of the simulator in the student’s sample.

**Figure 7:**
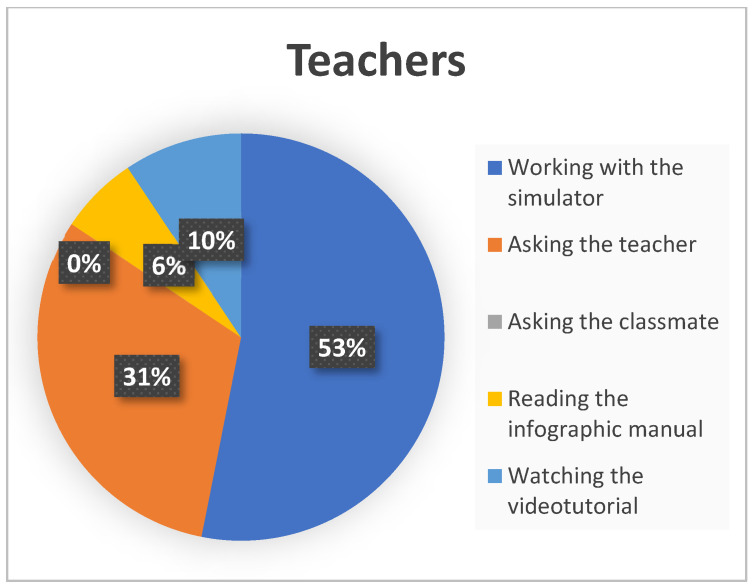
Percentages of the most effective strategies for knowledge/learning of the simulator in the teacher´s sample.

Finally, Figure 6 and Figure 7 show, together, the assessment of students and teachers about which strategies they considered the most effective for the knowledge/learning of the complex simulator.

In the comparative analysis it can be observed that the work with the simulator was the preferred strategy for both samples, followed by the questions to the teacher, also in both cases. Students threw equal ratings in percentage to the strategies of asking the teacher or a classmate and reading the manual. On the other hand, none of the respondent teachers considered the questions to a classmate valuable. The teachers´ sample also gave little importance to the study of graphic and audiovisual resources (6% and 9%).

## 4. Discussion

The learning domain best identified by students and teachers was the technical (operating the simulator at a basic level necessary to perform the exercise). That this domain is located above the theoretical domain and advanced management domain, speaks well of the intuitive nature of the friendly courseware and the interfaces of the simulators, but leaves us wishing for a deeper knowledge through a better use of the resources created.

As several studies by Bakr [14,16] suggest, knowledge of the simulator improves and encourages its use. This occurs especially when there are technical limitations, described from the first generated units [21] and which remain in the current units. These limitations are the selection of the rotating elements, the most pertinent management of the haptic field and the better integration of auditory, visual and haptic feedback. These factors improve with the explicit description of the management protocols.

At the same time, it is important to notice that the means, in none of the domains, reach high levels.

The lowest mean in the domains valued by the students was that of advanced management. In contrast, teachers considered that the theoretical domain of their students was lower than the advanced management.

This contradictory result supports the tendency to consider that skills are achieved intuitively, and it explains the teacher’s perception that the study of the manual is insufficient. Which, in our study, correlates significantly with low theoretical dominance.

Correlation analyses allow us to infer that the teachers tend to link the theoretical knowledge of the simulator with the study of the manual, while the technical domain is related to the study of the video, the latter being a powerful correlation of marked significance within the study.

One of the limitations of our research is the lack of previous studies on the management of Simodont^®^. Therefore, when it comes to contrasting the results obtained, we must rely on similar, but not identical, initiatives in medical education.

Bordes et al. [28] comment that the use of infographic and audiovisual resources has a great impact on active learning and student performance. This is due to its ability to focus on specific elements that are easy to assimilate. Although in their studies they do not compare the initiative with others, their results show a 79.7% viewing of short videos (between 5–10 min) and a visualization close to the figures of our study for the longer videos (between 45 min–1 h). In our research, we took into account the reduction of time and content to facilitate the assimilation and viewing of videos, given the numerous pieces of evidence that speak about it [25]. However, in the students’ responses, the weak correlation between the domains and the study of the resources indicates a tendency to not value well the prior technological preparation, which accuses a waste of the resources created and the probable increase of knowledge/learning of the simulators at the expense of the interaction with it, the day of the practice.

Students may consider that simulators are, by default, intuitive and prioritize preparing for the workshop at hand, rather than preparing to handle the simulator. This may be either due to lack of time or greater cognitive needs of other materials that need to be studied. In this way, it can be concluded that the knowledge of the new equipment is obtained more by trial and error than by previous preparation.

These affirmations would correspond with studies by McNulty et al. [29] when they state that students preferably turn to these materials if they feel they are having difficulties or when the resource offers a key content of the curriculum that they cannot access otherwise. We consider that, in our study, the student’s sense of trust with the simulator, the complementary nature of the didactic resources (not directly linked to the workshop content) and the certainty that they can rely on the teacher to resolve their doubts, explain the still insufficient reception of the material.

However, in the case of skills workshops with complex equipment, the lack of simulator information reduces the working time in the workshops as time is lost in learning how to operate the simulator. There may also be damage to the sensitive interfaces of these devices, damage to their haptic interfaces, loss of the courseware path and blockages of the units due to uncontrolled or arbitrary manipulation.

In the words of Roy et al. [30], the need to introduce state-of-the-art simulators has to be based on an in-depth knowledge of them. Plus, if, as posed by Boer et al. [31], most of the centers need confirmation that the new technology adds more value compared to the traditional method used, the impulse to introduce them in the programs will only start from the greater knowledge/learning of the simulator that the participants have.

At this point, it is important to raise a nuance. Although in this study the advanced management of the simulator qualifies the autonomy of the student in practical exercises assisted by teachers, it must be considered that the hardware of the simulators is constantly updated and integrates new elements of management, measurement and self-evaluation [17]. This constant update of the software needs to be considered as an important factor for the achievement of an advanced management.

It is also an inference from our study that the low perception of the study given by teachers of the informative written manual and the video tutorials respond to the certainty of their little use by students.

These results connect, within this same research, with descriptive analyses that indicate that both students and teachers consider that the best strategy for learning is the direct exchange with the simulator.

Undoubtedly, the direct exchange with the learning tool is a milestone for all in-depth knowledge of the technological genres [32]. However, in the case of the teaching activity, anticipation and training background are necessary to make the most of practice time, technical and human resources, protect equipment and create a training culture in which the learner is the protagonist. The prevalence of traditional concepts such as dependence on the teacher and asking the teacher questions to solve setbacks, raise the need to look for more innovative training solutions. These training solutions must be supported by technology, teamwork, and the linking of face-to-face activities with e-learning resources in a dynamic way and with more powerful intrinsic and extrinsic motivations.

## 5. Conclusions

Both students and teachers considered that the technical domain (to operate the simulator at a basic level necessary to perform the exercise) was the one that prevailed the most, which speaks favorably of the intuitive and friendly interface of the simulators. However, this level of domain may not be enough for a correct use of the simulator.

There was a tendency for students not to value highly the prior technological preparation. The study of the didactic resources created in the study did not reach the desirable high levels.

Both students and teachers considered that the best knowledge/learning strategy was the direct exchange with the simulator. However, in the teaching activities that concern us, anticipation and the training background are necessary to make the most of the practice times, human and technical resources, to protect the equipment and to create a training culture in which the student has the leading role.

## Figures and Tables

**Table 1:** Description of dependent variables.

Variable	Definition	Tool
Theoretical domain	Know how the simulator works at a conceptual or theoretical level, which does not imply putting it into function. Low level of knowledge/learning.	Polytomous closed questions on the student’s perception of knowledge/learning in use of the simulator. Qualified in 3 levels: 1. Never 2. Sometimes 3 Always Applied to students and teachers.
Technical domain	Operate the simulator to the basic level needed to perform the exercise. Medium level of knowledge/learning.	
Advanced management domain	Carry out the exercise autonomously and smoothly. High level of knowledge/learning.	

**Table 2:** Description of independent variables.

Variable	Definition	Tool
Study of the informative written manual	Know the level of assimilation of the resource through its use	Polytomous closed questions about the study of the resource. Qualified in 4 levels: 1. Unread/unviewed 2. 30% read/viewed (Low Level). 3. 50% read/viewed (Medium Level). 4. 100% read/viewed (High Level). Applied to students and teachers.
Study of the video tutorial		

**Table 3:** Perception of knowledge/learning of students (according to the students themselves).

		Theoretical Domain	Technical Domain	Advanced Management	Study of the Manual	Video Study
***n***	valid	175	175	175	175	175
	lost	0	0	0	0	0
Mean		2.34	2.47	2.09	3.43	3.22
Standard deviation		0.623	0.668	0.453	0.906	1.134

**Table 4:** Perception of knowledge/learning of students (according to teachers’ perception).

		Theoretical Domain	Technical Domain	Advanced Management	Study of the Manual	Video Study
***n***	valid	32	32	32	32	32
	lost	0	0	0	0	0
Mean		1.97	2.50	2.31	2.84	3.34
Standard deviation		0.400	0.672	0.471	0.515	0.787

**Table 5:** Correlations between simulator knowledge/learning variables and resources. Students sample.

			Theoretical Domain	Technical Domain	Advanced Management	Study of the Manual	Video Study
Spearman’s Rho coefficient	Theoretical Domain	Correlation coefficient	1.000	0.293 **	0.293 **	−0.015	−0.002
		Sig. (bilateral)	.	0.000	0.000	0.843	0.975
		n	175	175	175	175	175
	Technical Domain	Correlation coefficient	0.29 **	1.000	0.156 *	0.122	0.077
		Sig. (bilateral)	0.000	.	0.039	0.107	0.308
		n	175	175	175	175	175
	Advanced Management Domain	Correlation coefficient	0.293 **	0.156 *	1.000	−0.059	0.008
		Sig. (bilateral)	0.000	0.039	.	0.436	0.912
		n	175	175	175	175	175
	Study of the manual	Correlation coefficient	−0.015	0.122	−0.059	1.000	0.252 **
		Sig. (bilateral)	0.843	0.107	0.436	.	0.001
		n	175	175	175	175	175
	Study of the video tutorial	Correlation coefficient	−0.002	0.077	0.008	0.252 **	1.000
		Sig. (bilateral)	0.975	0.308	0.912	0.001	.
		n	175	175	175	175	175

* The correlation is significant at level 0.05 (bilateral). ** The correlation is significant at level 0.01 (bilateral).

**Table 6:** Correlations between simulator knowledge/learning variables and resources. Teachers sample.

			Theoretical Domain	Technical Domain	Advanced Management	Study of the Manual	Video Study
Spearman’s Rho coefficient	Theoretical Domain	Correlation coefficient	1.000	0.365 *	0.393 *	0.570 **	0.370 *
		Sig. (bilateral)	.	0.040	0.026	0.001	0.037
		n	32	32	32	32	32
	Technical Domain	Correlation coefficient	0.365 *	1.000	0.544 **	0.123	0.901 **
		Sig. (bilateral)	0.040	.	0.001	0.503	0.000
		n	32	32	32	32	32
	Advanced Management	Correlation coefficient	0.393 *	0.544 **	1.000	0.186	0.499 **
		Sig. (bilateral)	0.026	0.001	.	0.309	0.004
		n	32	32	32	32	32
	Study of the manual	Correlation coefficient	0.570 **	0.123	0.186	1.000	0.108
		Sig. (bilateral)	0.001	0.503	0.309	.	0.556
		n	32	32	32	32	32
	Study of the video tutorial	Correlation coefficient	0.370 *	0.901 **	0.499 **	0.108	1.000
		Sig. (bilateral)	0.037	0.000	0.004	0.556	.
		n	32	32	32	32	32

* The correlation is significant at level 0.05 (bilateral). ** The correlation is significant at level 0.01 (bilateral).

## Data Availability

Not applicable.
